# Clinical comments related to medullary thyroid cancer diagnosis and management

**DOI:** 10.1186/1756-6614-6-S1-S6

**Published:** 2013-03-14

**Authors:** Leonidas H Duntas

**Affiliations:** 1 Unit of Endocrinology, Diabetes and Metabolism, Evgenidion Hospital, University of Athens Medical School, 20 Papadiamantopoulou St., 115 28 Athens, Greece

## Abstract

**Background:**

The American Thyroid Association (ATA) and more recently the European Thyroid Association (ETA) Guidelines on diagnosis and treatment of medullary thyroid carcinoma (MTC) have provided an excellent tool which was formerly lacking in the field of management of MTC. However, some relevant clinical questions, as the use of somatostatin analogues in the treatment of MTC and the management of pregnant patients with MTC, which were recommended in the guidelines, have been lately extensively revised. Moreover the current issue whether GLP-1 (a glucagon-like peptide-1) analogue is associated with MTC has only superficially been analyzed.

**Methods:**

Publications have been retrieved in MEDLINE at Pubmed (there is no fix date retrospectively) up to October 2012 using the terms “medullary thyroid carcinoma”, “somatostatin”, “pregnancy” and “incretins”. The recommendations made by ATA and ETA were considered.

**Conclusions:**

There are no data supporting the application of somatostatin analogues in the treatment of MTC, while thyroid cancer during or after pregnancy has no impact on the prognosis of disease or on the outcome of pregnancy. However, women with MEN 2 should be carefully controlled before any planned or during any unplanned pregnancy. In contrast to animal studies, there are no consistent human data supporting a stimulatory effect of GLP-1 receptor activation by liraglutide, an incretin mimetic, on calcitonin levels, though establishment of a registry and further studies are required to exclude any association between GLP-1 analogue and MTC.

## Background

MTC, which accounts for 5-8% of all thyroid cancers, is mainly sporadic in nature, while a hereditary pattern (multiple endocrine neoplasia type 2 (MEN 2), transmitted as an autosomal-dominant trait due to germline mutations of the RET proto-oncogene, accounts for about 29% of cases [1].

In 2009, at the ETA annual meeting in Lisbon, the European Thyroid Association-Cancer Research Network (ETA-CRN) extensively commented on the American Thyroid Association Guidelines regarding the diagnosis and treatment of medullary thyroid cancer (MTC) published earlier that year [2]. These, in conjunction with the 2011 European Thyroid Association (ETA) Guidelines [3], succeeded in compensating for the previous lack of consensus on the management of this rare disease and now present a platform for optimal diagnostic and therapeutic approach to MTC, one which moreover demands multidisciplinary engagement. Some of the above mentioned comments, updated by references to current publications and expanded by several emerging findings, are highlighted in the present article which seeks to offer a broader perspective concerning certain clinical queries and the recommendations made.

## Clinical questions

### An oft-posed query: might somatostatin analogues be applicable as antitumor agents in MTC?

MTC is derived from the neural crest and exhibits a widely variable expression of somatostatin receptors which have been used in the detection of disease. Initially octreotide and recently 68Ga DOTA-peptides PET have been successfully employed for the detection of MTC neuroendocrine tumors [4]. More recent developments have provided encouraging results in imaging modalities of MTC, such as PET/CT with beta-emitter labeled DOTANOC, characterized by great affinity to the somatostatin receptors [5]. However, (18)F-DOPA PET/CT seems to be the most valuable imaging method in detecting recurrent MTC [6]. The optimal treatment of MTC is total thyroidectomy and removal of any metastatic tissue from the cervical region. Thus, any alternative treatment should be evaluated on an individual basis.

There are no randomized control studies but only case series or unsystematically conducted studies which were not able to demonstrate any consistent antitumor somatostatin effect. All studies (Table 1), which in total included 55 patients, showed subjective and biological partial remission in one third and in one fourth of the MTC patients, respectively, but no improvement in the natural course of the tumor [7]. Somatostatin analogues, i.e. octreotide or lanreotide, were administered at a dose of 200-1500 micrograms. Despite an initial decrease of calcitonin (Ct) and/of carcinoembryonic antigen (CEA) levels of up to 68% of the basal levels, there was a rebound within a few weeks to 84% and 105%, respectively, of the pretreatment values, thus indicating tachyphylaxis [7,8]. However, in another study a continuous effect on Ct was observed in two patients with MEN IIa treated with octreotide at a dose 1.5-2 mg/day [9]. More evident was an improvement in clinical symptoms as diarrhea, malaise and weight loss in all the patients [9]. Nevertheless, the results are notably heterogeneous, such as in a study including 18 patients where a greater than 20% decrease of Ct was apparently dependent on the dose of octreotide and the extent of disease (10). In contrast, different formulations of octreotide and lanreotide did not modify Ct and CEA levels in five patients with recurrent MTC treated over a period of 12 weeks [11]. In cases of advanced MTC with ectopic corticotropin-releasing hormone (CRH) and adrenocorticotropic hormone (ACTH) production leading to Cushing syndrome, the use of somatostatin analogues, especially when combined with interferon-alpha, improved diarrhea only transitorily in a few patients [12].

**Table 1:** Evaluation of somatostatin analogues in advanced medullary thyroid cancer.

Reference	Study	Treatment / Duration	No. of patients	Outcome
Vainas I et al. 2004	OS	Octreotide-(LAR)/ 21 months	22	No improvement of the natural course
Diez IJ, Iglesias P 2002	OS	Octreotide / Lanreotide / 12 weeks	5	No effect
Frank-Raue K et al. 1993	OS	Octreotide	7	No effect No beneficial effect on diarrhea
Mahler C et al. 1990	CR	Octreotide / 17 months	1 2	Tachyphylaxis
Modigliani E et al. 1989	OS	Octreotide / 37 days	18	Improvement of diarrhea in 2/9

OS: observational study, CR: control study

In summary, somatostatin analogues are ineffective in controlling tumor growth and are not recommended by ATA and ETA Guidelines as antitumor agents in MTC.

Basing their assessments on the currently available evidence, both the ATA and ETA Guidelines have recommended against the use of somatostatin analogues in MTC: Grade F (Rec. 84) [2] and Grade 2 and Quality of evidence + [3].

### Does medullary thyroid cancer compromise pregnancy?

There are only isolated healthcare reports of management of thyroid cancer (TC) discovered during or after pregnancy and TC does not appear to have a significant impact on the prognosis of the disease. Nor do Ct levels compromise pregnancy. Increase of Ct levels without localization of the disease should not constitute an absolute contraindication of pregnancy. However, in the event that multiple endocrine neoplasia 2A (MEN 2A), which is characterized by MTC, adrenal pheochromocytoma and hyperparathyroidism due to specific RET proto-oncogene mutations, remains unrecognized during pregnancy, then both woman and progeny are at risk of a crisis and of RET mutation transmission, respectively [13].

A case of bilateral pheochromocytoma in a term-pregnant patient with a previous history of MTC was described [14]. The genetic study revealed a heterozygous mutation, c.1900T>C, in the RET proto-oncogene that confirmed the diagnosis of multiple endocrine neoplasia type 2A (MEN2A). Unfortunately, the tumors remained unrecognized and caused a crisis with fatal outcome for the mother during the postpartum period. This instance, though rare, nevertheless underlines the necessity of controlling pregnant patients with a history of MTC, since early detection in such cases is capable of saving the mother and the fetus.

The Guidelines recommend screening for pheochromocytoma in women with RET mutation associated with MEN 2 before a planned pregnancy or at the beginning of an unplanned pregnancy: Grade B (Recommendation 25) [2].

### Do glucagon-like peptide-1 analogues increase the incidence of MTC?

Studies in rodents showed that the antidiabetic agent liraglutide, a glucagon-like peptide-1 (GLP-1) receptor agonist, increased risk of thyroid C-cell focal hyperplasia, a state which in rodents is considered as a preneoplastic lesion resulting in MTC, and C-cell tumors [15]. In humans, increases of calcitonin in patients injecting the incretin mimetic liraglutide, albeit falling within the normal range, were observed. Despite the acknowledged limitations to extrapolating findings from animal studies to humans, the results in rodents may possibly indicate a low risk for humans (14). Recently, a study was designed to determine whether GLP-1 receptor activation by liraglutide over a period of two years has an impact on Ct levels in type 2 diabetes mellitus and nondiabetes obese controls [16]. There was no consistent dose or time-dependent relationship between treatment groups, this suggesting that GLP-1 receptor activation has no effect on c-cell activity. However, as has been stated by the FDA, a cancer registry to monitor the annual incidence of MTC over the next 15 years is required in order to clarify any possible association between liraglutide and MTC.

## List of abbreviations used

ACTH: adrenocorticotropic hormone; CEA: carcinoembryonic antigen; CRH: corticotropin-releasing hormone; Ct: calcitonin; DT: doubling time; GLP-1: glucagon-like peptide-1; MEN2A: multiple endocrine neoplasia type 2A; MTC: medullary thyroid carcinoma.

## Competing interests

No competing interests are declared by the author of this paper.

## Declarations

This article has been published as part of *Thyroid Research* Volume 6 Supplement 1, 2013: European comments on Medullary Thyroid Cancer Management Guidelines of the American Thyroid Association. The full contents of the supplement are available online at http://www.thyroidresearchjournal.com/supplements/6/S1. Publication of this supplement has been supported by the European Thyroid Association-Cancer Research Network.
